# The Trend of Age-Group Effect on Prognosis in Differentiated Thyroid Cancer

**DOI:** 10.1038/srep27086

**Published:** 2016-06-08

**Authors:** Rong-liang Shi, Ning Qu, Tian Liao, Wen-jun Wei, Yu-Long Wang, Qing-hai Ji

**Affiliations:** 1 Department of Head and Neck Surgery, Fudan University Shanghai Cancer Center; Department of Oncology, Shanghai Medical College, Fudan University, Shanghai 200032, China; 2 Department of General surgery, Minhang Hospital, Fudan University, Shanghai 201199, China

## Abstract

Age has been included in various prognostic scoring systems for differentiated thyroid cancer (DTC). The aim of this study is to re-examine the relationship between age and prognosis by using Surveillance, Epidemiology, and End Results (SEER) population-based database. We identified 51,061 DTC patients between 2004 and 2012. Patients were separated into 10-year age groups. Cancer cause-specific survival (CSS) and overall survival (OS) data were obtained. Kaplan-Meier and multivariable Cox models were built to analyze the outcomes and risk factors. Increasing age gradient with a 10-year interval was associated with the trend of higher proportions for male gender, grade III/IV and summary stage of distant metastases. Both CSS and OS continued to worsen with increasing age, being poorest in in the oldest age group (≥71); multivariate analysis confirmed that CSS continued to fall with each age decade, significantly starting at 60 years (HR = 7.5, 95% 1.0–54.1, *p* = 0.047) compared to the young group (≤20). Similarly, multivariate analysis suggested that OS continued worsening with increasing age, but starting at 40 years (HR = 3.7, 95% 1.4–10.1, *p* = 0.009) compared to the young group. The current study suggests that an age exceeding 60 years itself represents an unfavorable prognostic factor and high risk for cancer-specific death in DTC.

Thyroid cancer is the most common endocrine malignancy. With the annually increasing number, approximately 62,980 estimated thyroid cancers are newly diagnosed in the United States in 2014^1^, more than 90% of which are differentiated thyroid cancers (DTC). DTC usually is subdivided into papillary thyroid cancer (PTC) and follicular thyroid cancer (FTC). Although it has been reported that DTC presents a relatively excellent prognosis previously^2^, about 10% of the patients still die of this cancer^3^,^4^. Therefore, different scoring systems have been created in DTC for prognosis stratification aiming to identify an optimal therapy for high-risk patients to reduce disease recurrence^5^,^6^. Age, in particular, is considered to be the most important prognostic factor as demonstrated by the majority of current risk stratification systems^7^,^8^. The new tumor-node-metastasis(TNM)classification^9^,^10^ is to our knowledge one of the most common systems adopting an age cutoff of 45 years to stratify the patients in high- and low-risk groups for cancer-specific mortality. Conversely, a series of different setups such as EORTC(1979), AGES (1987), AMES (1988), DAMES (1992), MACIS (1993) consider age as a continuous factor that affecting the prognosis without any stratification^11^,^12^. The controversies on how to categorize ages for predicting prognosis in DTC derive from several studies suggest the relationship between the age and mortality is represented by an exponential function rather than a categorical variable^13^. The present study is to elucidate the prognostic implication of age in patients with DTC as reported by the SEER Program from 2004 to 2012. Specifically, we compared the survival of patients with different age ranges aiming to accurately determine the relationship between age and outcome of DTC patients.

## Methods

We extracted data from the SEER cancer registry to conduct this study. SEER, a population-based registry sponsored by the National Cancer Institute, collects information on cancer incidence and survival from 17 population-based cancer registries, including approximately 28% of the U.S. population^14^. SEER data contains no identifiers and is publicly available for studies on cancer-based epidemiology and health policy. The National Cancer Institute’s SEER*Stat software (Surveillance Research Program, National Cancer Institute SEER*Stat software, www.seer.cancer.gov/seerstat Version 8.1.2) was used to identify patients whose pathological diagnosis was DTC between 2004 and 2012. Patients with surgical therapy for DTC as the only malignancy or the first one of more malignancies were included. Histology types were limited to papillary carcinoma (8050/3, 8052/3, 8340/3, 8341/3, 8342/3, 8343/3, 8344/3, 8347/3) and follicular adenocarcinoma (8330/3, 8331/3, 8332/3, 8335/3, 8346/3). The histology classification is derived from ICD-0-3 SEER site/histology validation list (2015). Other patients were excluded.

### Ethics statement

This study was in compliance with the Helsinki Declaration. An independent ethics committee/institutional review board at Fudan University Shanghai Cancer Center approved our study. The methods were carried out in accordance with the approved guidelines in this study. Data released from the SEER database do not require informed patient consent because they contain no identifiers and were publicly available. We have got permission to access the research data file in the SEER program by National Cancer Institute, USA and the reference number was 13579-Nov 2014.

### Clinicopathological variable assessment and statistical analysis

The variable of “age” refers to “age at diagnosis” when not otherwise specified. Young patients (≤20 yr) with DTC were grouped together because there was a relatively small number, the rest of the patients were stratified into 10-year age groups. Compared to dichotomizing patients into younger (<45 yr) *vs.* older group (≥45 yr), the application of 10-year age ranges allowed for a more detailed analysis of clinicopathologic features and treatment by age. The endpoint of the present study was DTC cause-specific survival (CSS) which was calculated from the day of diagnosis to the day of cancer-specific death and was shown as “SEER cause-specific survival” in the SEER database. Overall survival (OS) was calculated from the day of diagnosis to the day of death, which was indicated as “Vital Status” in the SEER database. Race, sex, tumor grade, histological type, summary stage, TNM stage, radiation, CSS and OS were assessed. We followed the guidance of the 2010 TNM classification of American Joint Committee on Cancer/International Union Against Cancer (AJCC/UICC)^9^,^10^.

Chi-square (χ2) test was used to evaluate the independent variables. Survival rate was generated using Kaplan-Meier curve, and the differences were compared with the log-rank test. Multivariate Cox regression models were used for analysis of risk factors of survival. The 95% confidence intervals (CIs) for proportions were calculated. The nonlinear effect of age on the hazard ratio (HR) of DTC-specific mortality was assessed using quintic polynomial regression, with the R^2^ reported. *P* < 0.05 was considered statistically significantly. Statistical analysis was performed using SPSS for Windows 13.0 computer software (SPSS Inc., Chicago, IL).

## Results

### Baseline characteristics and comparison among groups according to age

**Figure 1:**
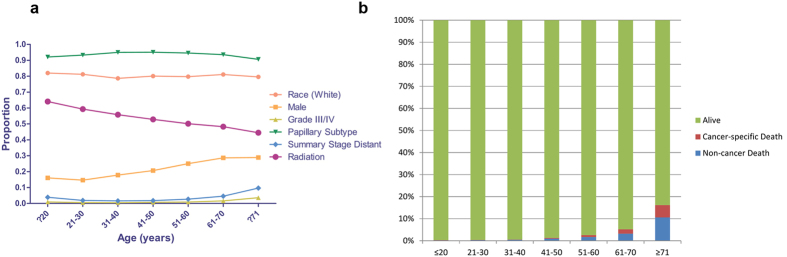
The comparisons of baseline characteristics according to age in patients with differentiated thyroid cancer. (**a**) The variations of proportions for clinicopathologic features in each age group. (**b**) The proportions of death events in each age group.

We identified 51,061 eligible patients with DTC in SEER database between 2004 and 2012. There were 1,382 patients in the young (≤20) group that representing the smallest number of subjects; the majority of patients was classified into 31–40 (n = 10,207), 41–50 (n = 12,624), and 51–60 (n = 11,237) groups (Table 1). The proportions of races and papillary subtype in each group were comparable with slight variations (Table 1, Fig. 1a). The proportion of patients receiving radiation gradually decreased with age, however, the proportion of risk factors such as male gender, grade III/IV and summary stage of distant had increasing trend in the entire cohort (Table 1, Fig. 1a). The overall death events increased from 0.3% in the young group to 16.2% in the oldest group and this phenomenon was also observed for cancer specific deaths within overall death events (Table 1, Fig. 1b).

### The impact of age on survival in DTC

**Figure 2:**
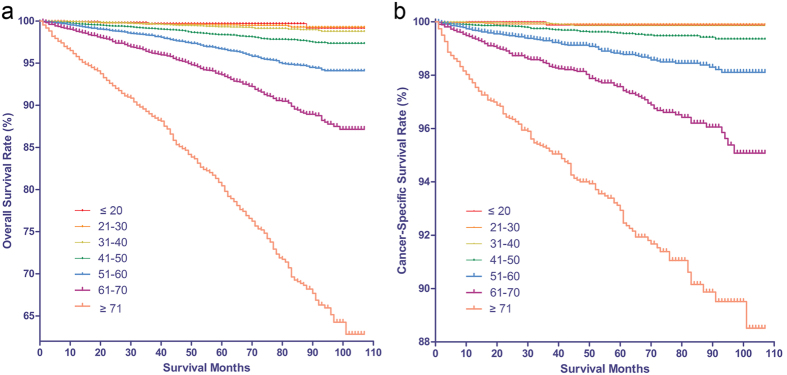
The Kaplan Meier curves for survival in patients with differentiated thyroid cancer (DTC) according to age. (**a**) The Kaplan Meier curves for overall survival in patients with DTC according to the increasing age gradient. (**b**) The Kaplan Meier curves for cancer-specific survival in patients with DTC according to the the increasing age gradient.

**Figure 3:**
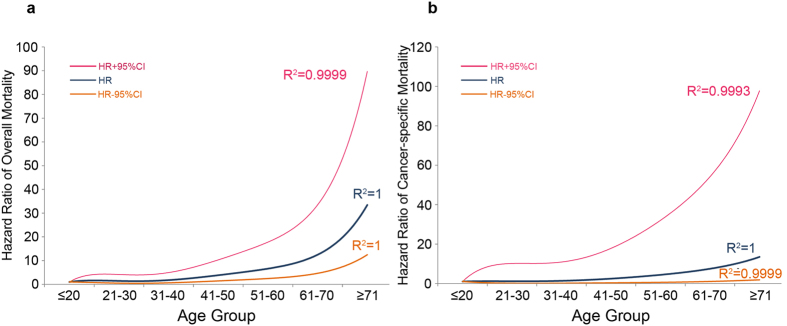
Estimates of hazard ratios (HRs) of overall (**a**) and cancer-specific (**b**) mortality changing with age for patients with differentiated thyroid cancer using quintic polynomial regression. The solid blue lines represent the estimates of HRs, whereas the dotted orange lines represent the 95% confidence intervals. All R^2^ values are reported.

The mean length of follow-up was 45.7 months (SD = 30.5), with a range of 0–107 months. The OS and CSS rate was 96.5% and 98.8% at 5 years, 93.8% and 98.1% at 8 years from the time of diagnosis. Although patients with DTC showed relatively favorable prognosis, we observed that there was continued worsening in OS and CSS rates with increasing age by Kaplan–Meier curves, both being poorest in elderly(≥71) groups (Fig. 2a,b). The results of the univariate Cox proportional hazard regression analysis investigating the differences in survival rates among groups confirmed the worsening trends of OS and CSS with respect to aging (Table 2). To determine how strongly increasing age was associated with mortality relative to other known risk factors in DTC, we performed multivariate Cox regression analysis. The risk of mortality increased with increasing age: on the one hand, patients in the 41–50, 51–60, 61–70 and ≥71 groups exhibited a significantly greater risk of overall mortality than the young group; on the other hand with respect to cancer-specific mortality, higher risk began to be observed until age older than 60. The risk of cancer-specific death was significantly higher in 61–70 and ≥71 age group, who were about 7.5 and 13.5 times more likely to die of cancer than young (≤20) age group. The plots of HRs in subgroups according to different age ranges showed that hook-shaped curves and HRs started to apparently increase in the above 40 years age group for OS and 60 years age group for CSS (Fig. 3a,b). An age of 60 years was tested by performing receiver operating characteristic (ROC) analysis for a meaningful separation of predicting cancer-specific death, the result suggested that an area under the curve was 0.831 (*p* = 0.001, 95% CI 0.815–0.848) with the sensitivity (67.1%) and specificity (80.9%).

### The predictability of age on prognosis in histologic subtypes of thyroid cancer

**Figure 4:**
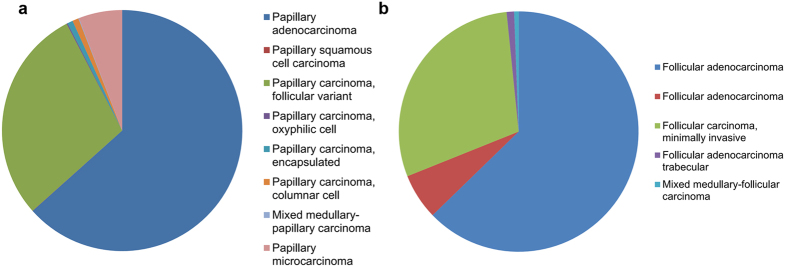
The compositions of histologic subtypes for papillary thyroid cancer (**a**) and follicular thyroid cancer (**b**).

Of note, this series comprised of a dominant proportion of papillary thyroid cancer (PTC, 94.2%) and a low proportion of follicular thyroid cancer (FTC, 5.8%). The composition of histologic subtypes for PTC and FTC were shown in Fig. 4a,b. In PTC and FTC patients, the predominating histologic variants were follicular variant and minimally invasive, respectively. The variants considered to carry a poorer prognosis such as solid, tall-cell, and poorly differentiated were rare in this series, which was different from other systems incorporating histologic variants in the prognostic model^15^,^16^. Although PTC and FTC were staged in the same way in current systems, the results of univariate analysis demonstrated highly significant differences of death risks between the different groups (PTC *vs.* FTC, Table 2). Therefore, we tested the predictability of age on prognosis in subtypes of DTC, respectively. Similarly, increasing was associated with the worsening trends of cancer-specific survivals in both groups. However, the trends of decreasing CSS were not statistically significant among the different age groups of patients with PTC, or patients with FTC until they were older than 70 years (Supplemental Fig. 1a,b).

### The other risk factors associated with mortality in DTC

In multivariate analysis, the results also suggested a series of factors that were previously demonstrated to be important in predicting poor prognosis in DTC patients, such as male gender, higher tumor grade (III/IV) and cancer stage (Table 2). In particular, the adjuvant radiation therapy had improved the OS but not CSS rate in the entire cohort according to the multivariate analysis (Table 2).

## Discussion

Thyroid cancer, especially DTC, has presented an obviously increasing incidence all over the world. Due to the excellent post-treatment outcome, it is difficult to make a randomized clinical trial to study these cancers. Most current staging evaluating the risk of cancer-specific death in DTC are developed by multivariate analysis of a specified patient population, there are many controversies when applying a certain system to a different patient population^17^. Age at diagnosis is considered to be one of the established risk factors for stratification^18^,^19^,^20^, however, the rationale for how to define the relationship between mortality and age needed to be clarified in DTC. In the present study, we divided DTC patients into subgroups with a 10-year intervals to assess the differences of clinicopathologic features and oncological outcomes among them; we found that the increasing age was associated with the high proportions of risk factors conferring unfavorable prognosis (such as male gender, grade III/IV and summary stage of distant metastases) and high risk of death events referring to both overall and cancer-specific reasons. The survival analysis confirmed the results and suggested that CSS of patients with DTC increased significantly until they were elder than 60 yr compared to the young groups.

Although DTC affects young adults with a significantly increasing incidence occurring between ages 25–54 years, the incidence appears to be rising in older people (≥65 years) simultaneously^21^. Similar to unfavorable histopathological features, it has been demonstrated that old age is a predictive factor for CSS in patients with DTC. The results of the present study repeatedly indicated that OS and CSS deteriorated with aging in DTC patients, and extremely worst among elderly patients (>70 years). The increased aggressiveness with the age gradient corresponded to a variety of factors, such as higher proportion of male gender, advanced tumor grade, follicular subtype, advanced tumor stage (Summary Stage and AJCC 7^th^ Stage), which were all proved to be independent risk factors for prognosis in univariate analysis. Given the substantial diversity of clinicopathologic features among different age gradients, it has been suggested that DTC may develop “separate forms” in the progression of aging. Additionally, we found that the ratio of patients who received radiation therapy decreased with age, and it has been reported that the effectiveness of radioiodine therapy decreases in the elder group due to the fact that the uptake of radioiodine is age-dependent^22^. This may be another reason why DTC patients have deteriorated prognosis with aging.

Liang *et al.* reviewed 9 staging systems derived from DTC series, found that 5 of them included age as a contributor for risk classification^23^. Most of stratification systems considered DTC patients under a certain age cutoff as less risk than those who were older. For example, an age of 45 was the cutoff point for continuous variables in Memorial Sloan Kettering (Grade, Age, Metastasis, Extent, Size or GAMES) and AJCC/UICC systems^9^,^24^, while 50 years of age was applied in University of Alabama and M.D. Anderson (UAB&MDA) system^25^. Thus, there is a lack of consensus among the staging systems regarding to the age threshold to be adopted. On the basis of the present study, we found that young patients (≤20 yr) had a favorable CSS rate, compared to the young group, the DSS for different age groups did not show a statistically significant decrease until the age of 60 years when the first significant drop was observed. Multivariate analysis by the Cox proportional HR model confirmed that the importance of age as an independent risk determinant became evident only after the age of 60 years and the subset analysis of patients above 60 years showed obviously increased cancer-specific mortality compared to young group.

The finding that each age decade was independently associated with a worse prognosis starting at 60 years was similar to that of recent study^13^. This suggests that the mortality rate in patients younger than 60 years is better, and the currently practiced cut off age such as 45 or 50 year seems to be lower. The dominating proportion in this cohort for PTC (94.2%), which is the less invasive subtype of DTC compared with FTC^26^, and the high constitution of papillary thyroid microcarcinoma (PTMC, Fig. 4a) with indolent behaviors, may account for the reason why a great number of staging systems including the current TNM system intended for DTC used relatively lower cutoff ages for stratifying patients into different risk categories behavior^27^,^28^.

Interestingly, the OS rate continues to fall with the age gradient, but significantly starting at 40 years compared to the young group. Unlike young populations with thyroid disease, older patients often have multiple complex illnesses coincident with their thyroid disease, some of which may be lethal such as cardiovascular disease, respiratory disease and other malignant tumors^29^. Recognition of these disorders will lead to better evaluation and appropriate therapy in elderly patients.

Except for the consideration of age and other clinicopathologic factors such as male gender, higher tumor grade (III/IV) and cancer stage when evaluating the risk of mortality in DTC, current studies suggest that complete surgical resection and postoperative radiation therapy are comparatively significant prognostic factor, especially in high risk patients^30^. Unfortunately, the details on surgical and radiation therapies were incomplete in the process of using the SEER database for survival analysis. However, given the aggressive features and decreased prognosis associated with aging, the appropriate surgery for initial management is of great importance for the elderly patients. To find out whether treatment could possibly affect the relative predictability of prognosis in different age patients, a comparative study with enough length of follow-up is necessary to be conducted based on subgroup analysis including relatively homogeneous DTC patients but with different procedures of thyroidectomy followed by radiation therapy or not.

In light of the results from present study, we found DTC with aging generally showed more aggressive features and decreased prognosis compared with younger adults. After the age of 60 years, the CSS was found to fall significantly in the older patients. Since the influence of age on prognosis is affirmed, clinicians who see older patients with DTC need to be aware of their poorer prognosis, especially older than 60 years.

## Additional Information

**How to cite this article**: Shi, R.-L. *et al.* The Trend of Age-Group Effect on Prognosis in Differentiated Thyroid Cancer. *Sci. Rep.* **6**, 27086; doi: 10.1038/srep27086 (2016).

## Supplementary Material

Supplementary Information

## Figures and Tables

**Table 1:** Characteristics of DTCs from SEER Database by Age.

	≤20	21–30	31–40	41–50	51–60	61–70	≥71	P value
Variable	(n = 1382)	(n = 5626)	(n = 10207)	(n = 12624)	(n = 11237)	(n = 6492)	(n = 3493)	
Race								
Black	54	242	485	702	648	343	177	0.001
%	3.9	4.3	4.8	5.6	5.8	5.3	5.1	
White	1133	4569	8031	10112	8957	5263	2779	
%	82.0	82.1	78.7	80.1	79.7	81.1	79.6	
Other^1^	172	715	1540	1650	1493	815	512	
%	12.4	12.7	15.1	13.1	13.3	12.6	14.7	
Unknown	23	100	151	160	139	71	25	
%	1.7	1.8	1.5	1.3	1.2	1.1	0.7	
Sex								
Male	222	824	1822	2605	2813	1861	1009	0.001
%	16.1	14.6	17.9	20.6	25.0	28.7	28.9	
Female	1160	4802	8385	10019	8424	4631	2482	
%	83.9	85.4	82.1	79.4	75.0	71.3	71.1	
Grade^2^								
I/II	270	1151	1946	2313	1985	1132	585	0.001
%	19.5	20.5	19.1	18.3	17.7	17.4	16.7	
III/IV	13	30	50	92	96	105	126	
%	0.9	0.5	0.5	0.7	0.9	1.6	3.6	
Unknown	1099	4445	8211	10219	9156	5255	2782	
%	79.5	79.0	80.4	80.9	81.5	80.9	79.6	
Histology								
Papillary	1273	5247	9693	12005	10628	6074	3166	0.001
%	92.1	93.3	95.0	95.1	94.6	93.6	90.6	
Follicular	109	379	514	619	609	418	327	
%	7.9	6.7	5.0	4.9	5.4	6.4	9.4	
Summary Stage								
Regional/ Localized	1325	5497	10016	12377	10908	6181	3138	0.001
%	95.9	97.7	98.1	98.0	97.1	95.2	89.8	
Distant	54	107	163	222	304	295	337	
%	3.9	1.9	1.6	1.8	2.7	4.5	9.6	
Unknown	3	22	28	25	25	16	18	
%	0.2	0.4	0.3	0.2	0.2	0.2	0.5	
AJCC 7^th^ TNM Stage								
I/II	1355	5532	10051	9888	7328	4217	1946	0.001
%	98.0	98.3	98.5	78.3	65.2	65.0	55.7	
III/IV	–	–	–	2231	3303	1916	1315	
%	–	–	–	17.7	29.4	29.5	37.6	
Unclassified	27	94	156	505	606	359	232	
%	2.0	1.7	1.5	4.0	5.4	5.5	6.6	
Radiation^3^								
Yes	886	3338	5699	6671	5633	3134	1553	0.001
%	64.1	59.3	55.8	52.8	50.1	48.3	44.5	
No	469	2174	4347	5778	5414	3270	1878	
%	33.9	38.6	42.6	45.8	48.2	50.4	53.8	
Unknown	27	114	161	175	190	88	62	
%	2.0	2.0	1.6	1.4	1.7	1.4	1.8	
Vital Status								
Overall Death	4	22	54	164	292	338	565	0.001
%	0.3	0.4	0.5	1.3	2.6	5.2	16.2	
Alive	1378	5604	10153	12460	10945	6154	2928	
%	99.7	99.6	99.5	98.7	97.4	94.8	83.8	

^1^Including American Indian/AK Native, Asian/Pacific Islander.

^2^Grade I, well differentiated; Grade II, moderately differentiated; Grade III, poorly differentiated; Grade IV, undifferentiated; anaplastic.

^3^Including radioisotopes, radioactive implants, beam radiation, and combination of beam with implants or isotopes.

LGAbbreviations: DTC, differentiated thyroid cancer.

**Table 2:** Univariate and multivariate survival analyses of DTCs according to various clinicopathological variables.

Variable	No. Case	Overall Survival					Cancer-specific Survival				
		Univariate analysis (Unadjusted analysis)		Multivariate analysis (Adjusted analysis)			Univariate analysis (Unadjusted analysis)		Multivariate analysis (Adjusted analysis)		
		Log rank χ^2^ test	*P*value	HR^4^	95%CI	*P* value	Log rank χ^2^ test	*P*value	HR^5^	95%CI	*P* value
Race											
Black	2651	17.864	0.001	1.0	Reference		17.488	0.001	1.0	Reference	
White	40844			0.7	00.6–0.9	0.0–01			1.1	0.7–1.7	0.797
Other^1^	6897			0.6	0.5–0.8	0.001			1.1	0.7–1.8	0.773
Unknown	669			0.2	0.1–0.7	0.007			–	–	–
Sex											
Male	11156	264.902	0.001	1.0	Reference		120.124	0.001	1.0	Reference	
Female	39905			0.6	0.6–0.7	0.001			0.7	0.6–0.9	0.002
Age											
≤20	1382	3188.966	0.001	1.0	Reference		1168.245	0.001	1.0	Reference	
21–30	5626			1.4	0.5–4.1	0.534			1.1	0.1–10.1	0.915
31–40	10207			1.9	0.7–5.1	0.232			1.4	0.2–11.1	0.748
41–50	12624			3.7	1.4–10.1	0.009			2.4	0.3–17.6	0.389
51–60	11237			6.7	2.5–18.0	0.001			4.4	0.6–32.2	0.140
61–70	6492			12.4	4.6–33.4	0.001			7.5	1.0–54.1	0.047
≥71	3493			33.4	12.5–89.7	0.001			13.5	1.9–97.8	0.010
Grade^2^											
I/II	9382	2082.423	0.001	1.0	Reference		4545.199	0.001	1.0	Reference	
III/IV	512			5.0	4.0–6.2	0.001			9.4	6.8–13.0	0.001
Unknown	41167			1.0	0.9–1.2	0.783			1.1	0.8–1.5	0.562
Histology											
Papillary	48086	47.526	0.001	1.0	Reference		46.145	0.001	1.0	Reference	
Follicular	2975			1.1	0.9–1.3	0.219			1.2	0.9–1.5	0.231
Summary Stage											
Regional/Localized	49442			1.0	Reference		4425.317	0.001	1.0	Reference	
Distant	1482			3.6	3.1–4.1	0.001			7.6	6.2–9.4	0.001
Unknown	137			1.3	0.6–2.8	0.508			4.8	2.0–11.4	0.001
AJCC 7^th^ Stage											
I/II	40317	1206.620	0.001	1.0	Reference		1378.796	0.001	1.0	Reference	
III/IV	8765			1.8	1.6–2.0	0.001			5.5	4.0–7.5	0.001
Unclassified	1979			1.9	1.5–2.3	0.001			4.8	3.2–7.2	0.001
Radiation^3^											
No	26914	2.761	0.251	1.0	Reference		48.602	0.001	1.0	Reference	
Yes	23330			1.3	1.1–1.4	0.001			0.9	0.7–1.1	0.232
Known	817			1.3	0.9–2.0	0.194			0.8	0.4–1.8	0.651

^1^Including American Indian/AK Native, Asian/Pacific Islander.

^2^Grade I, well differentiated; Grade II, moderately differentiated; Grade III, poorly differentiated; Grade IV, undifferentiated; anaplastic.

^3^Including radioisotopes, radioactive implants, beam radiation, and combination of beam with implants or isotopes.

^4^HR is presented as risk of overall mortality when specific groups are compared with reference groups.

^5^HR is presented as risk of cancer-specific mortality when specific groups are compared with reference groups.

Abbreviations: DTC, differentiated thyroid cancer; HR, hazard ratio; CI, confidence interval.
