# Supplementary material for: The Trend of Age-Group Effect on Prognosis in Differentiated Thyroid Cancer
Source: Sci Rep. 2016 Jun 8;6:27086. doi: 10.1038/srep27086 (PMC4897617; doi:10.1038/srep27086)

The Trend of Age-Period Effect on Prognosis in Differentiated Thyroid Cancer

Author names: Rong-liang Shi[†,1,2] M.D., Ning Qu[†,1] M.D., Tian Liao[1] Ph.D., Wen-jun Wei[1] M.D., Yu-Long Wang[*,1] M.D.Ph.D.,Qing-hai Ji[*,1] M.D.

Figure S1. The Kaplan Meier curves for cancer-specific survival in patients with papillary thyroid cancer (a) and follicular thyroid cancer (b) according to age.

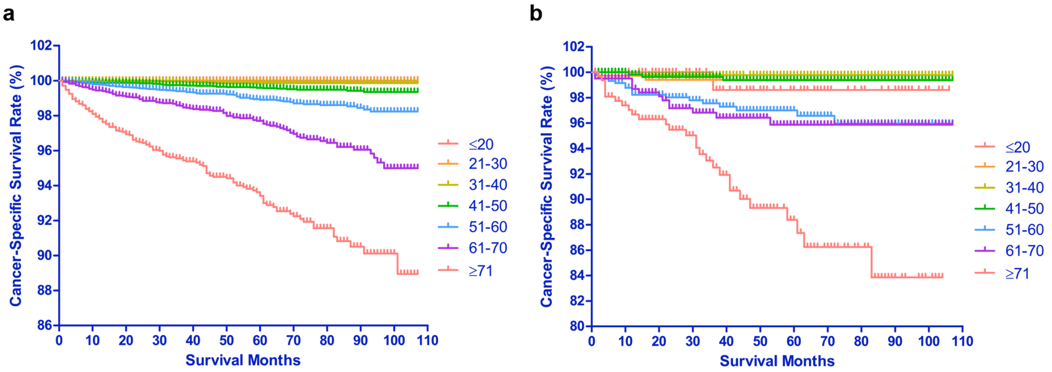

Supplement: Supplementary Information [file srep27086-s1.pdf]
